# Two Siblings With Recurrent Fevers: The Path to Mevalonate Kinase Deficiency Diagnosis

**DOI:** 10.7759/cureus.33613

**Published:** 2023-01-10

**Authors:** Joana Pereira-Nunes, Cristina Ferreras, Ana Grangeia, Francisca Aguiar, Mariana Rodrigues, Iva Brito

**Affiliations:** 1 Department of Pediatrics, Centro Hospitalar Universitário de São João, Porto, PRT; 2 Department of Gynecology-Obstetrics and Pediatrics, Faculty of Medicine of Porto University, Porto, PRT; 3 Department of Neonatology, Centro Hospitalar Universitário de São João, Porto, PRT; 4 Department of Medical Genetics, Centro Hospitalar Universitário de São João, Porto, PRT; 5 Department of Genetics, Faculty of Medicine of Porto University, Porto, PRT; 6 Pediatric and Young Adult Rheumatology Unit, Centro Hospitalar Universitário de São João, Porto, PRT; 7 Pediatric Medicine, Faculty of Medicine of Porto University, Porto, PRT

**Keywords:** urinary mevalonic acid, mevalonate kinase gene, immunoglobulin d, hyperimmunoglobulin d syndrome, autoinflammatory syndrome

## Abstract

Systemic autoinflammatory diseases (SAIDs) are a group of disorders that constitute a rare cause of recurrent fevers. Recurrent fevers are defined as periodic febrile episodes lasting from days to weeks, separated by symptom-free intervals of variable duration. They present multiple etiologies, representing a diagnostic challenge. Mevalonate kinase deficiency (MKD) is a genetic SAID, a rare hereditary recurrent fever syndrome (HRF) caused by pathogenic variants in the mevalonate kinase (MVK) gene. It is characterized by the early onset of periodic fever flares, frequently associated with joint, gastrointestinal, skin, and lymph node involvement. Although elevated serum immunoglobulin D (IgD) levels were previously considered an MKD's hallmark, normal values do not exclude it. High serum immunoglobulin A (IgA) is frequent. An acute-phase response and elevated urinary mevalonic acid (UAV) excretion during flares may aid in the diagnosis. Genetic testing is an essential tool to confirm the diagnosis. The authors report two siblings presenting with early infancy onset of recurrent febrile illness and characteristic associated symptoms, one of which was initially misdiagnosed with periodic fever, aphthous stomatitis, pharyngitis, and adenitis (PFAPA) syndrome. MKD diagnoses were only established at 12 and nine years old, respectively, after the identification of the same two MVKgene variants. The diagnosis in the eldest favored the earlier recognition of MKD in the youngest. Owing to its wide spectrum of manifestations, with many being nonspecific and/or shared with other more frequent entities, a significant proportion of MKD patients present a long delay until its final establishment. These cases illustrate the MKD diagnosis and management's difficulties, reinforcing the importance of a careful clinical history and HRF awareness for its prompt diagnosis and appropriate precocious referral.

## Introduction

Recurrent fevers are defined as periodic febrile episodes lasting from days to weeks, separated by symptom-free intervals of variable duration [1,2]. They present multiple possible etiologies (infectious, malignant, autoimmune, inflammatory, and genetic), representing a diagnostic challenge. Systemic autoinflammatory diseases (SAIDs) are a spectrum of disorders characterized by multisystemic immunodysregulation due to an exaggerated activation of the innate immune system in the absence of specific antigen-dependent mechanisms [1-3]. They are characterized by recurrent or continuous inflammation, constituting a rare cause of recurrent fevers [2,4].

Mevalonate kinase deficiency (MKD) is a genetic SAID, mediated by proinflammatory cytokine interleukin-1 (IL-1) [3]. It is a rare hereditary recurrent fever syndrome (HRF), with an incidence of less than 1/1.000.000 [5] and caused by pathogenic variants in the mevalonate kinase (MVK) gene, which results in variable degrees of mevalonate kinase enzyme deficiency [2,3,6]. MKD is characterized by the early onset of periodic fever flares, frequently associated with joint, gastrointestinal, skin, and lymph node involvement [6]. During flares, patients present an acute-phase response [6,7]. Although elevated serum immunoglobulin D (IgD) levels were previously considered MKD's hallmark, normal values do not exclude it [3,6,7]. High serum immunoglobulin A (IgA) is frequently present [6,7]. Elevated urinary mevalonic acid (UAV) excretion during flares may aid in the diagnosis and are more specific [2,3]. Genetic testing is an important tool to confirm the diagnosis [2,3].

We report two siblings presenting with recurrent fevers, one of which was initially misdiagnosed with periodic fever, aphthous stomatitis, pharyngitis, and adenitis (PFAPA) syndrome, in whom identification of the same two MVK gene variants confirmed MKD. They illustrate the diagnosis and management difficulties, reinforcing the importance of a careful clinical history and HRF awareness for its prompt diagnosis and appropriate precocious referral.

## Case presentation

Case one

An 18-year-old girl, the first daughter of non-consanguineous Caucasian parents, was first referred to our unit at the age of 12 with ongoing episodes of recurrent fever. At one month of age, she was hospitalized for suspected sepsis. Since then, she had experienced recurrent episodes characterized by high fevers, accompanied by oral ulcers, cervical lymphadenopathies, pharyngitis, and occasionally polyarthralgia. At first, these episodes occurred monthly and then at variable intervals, between one to six months, lasting for three to seven days with spontaneous resolution. She was otherwise asymptomatic, with normal inflammatory markers between flares, and presented adequate growth and neurodevelopment. Family history was unremarkable.

After excluding unusual infectious, malignant, and autoimmune etiologies, a PFAPA diagnosis was assumed by her attending general pediatrician. The patient was treated with a one to two day course of prednisolone with a favorable response. At the age of five, she underwent a tonsillectomy without any improvement.

At the age of 12, she presented *de novo* genital ulcers during a new febrile episode and was referred to our pediatric rheumatology unit due to Behçet's syndrome suspicion. Upon reviewing the history over the years, during attacks, the patient often presented a urticariform rash and abdominal pain with diarrhea; chest pain occurred rarely. Despite an improvement with steroids, the attack was not abruptly halted by it and low fevers persisted for a few days. It was also clear that immunizations and viral infections triggered febrile attacks. Combined with the early onset of episodes (clearly before 12 months), a SAID was suspected.

Extensive investigation during an asymptomatic period revealed normal erythrocyte sedimentation rate (ESR), C-reactive protein (CRP), and serum amyloid A (SAA). Serum immunoglobulins, including IgA, were in the normal range for age, except for a high IgD: IgA 163 mg/dL (N: 85-211) and IgD 60 mg/dL (N: <14 mg/dL). During attacks, laboratory findings showed elevated ESR, CRP, and SAA, reflecting an acute systemic inflammatory process. IgA and IgD were also elevated: IgA 371 mg/dL (N: 85-211) and IgD 77.4 mg/dL (N: <14 mg/dL). High UAV excretion was also detected: 31.9 µmol/mmol creatinine (N <0.3) (Table 1). Additional investigations were non-contributory. The clinical picture associated with high IgD, IgA, and UAV excretion led to a high degree of MKD suspicion. DNA analysis of the MVK gene identified two pathogenic variants in heterozygosity, c.79-2A>G and c.1129G>A (p.Val377Ile), confirming the diagnosis. Genetic screening carried out on the parents confirmed that the detected variants were in compound heterozygosity in the daughter.

**Table 1:** Laboratory results of cases one and two CRP - C-reactive protein; ESR - erythrocyte sedimentation rate; IgA - immunoglobulin A; IgD - immunoglobulin D; NA - not available; SAA - serum amyloid A;  UAV - urinary mevalonic acid

Laboratory results (units of measurement)	Reference values	Case one (female, 12 years old)		Case two (male, nine years old)	
		Between flares	During flares	Between flares	During flares
CRP (mg/L)	<20 mg/L	5	180.0	3	153.5
ESR (mm/h)	0-20 mm/h	7	47	13	54
IgA (mg/dL)	[9-12[ years old: 71-191 [12-16[ years old: 85-211	163	371	516	602
IgD (mg/dL)	<14	60.0	77.4	<0.5	45.0
SAA (mg/dL)	< 6.4	5.2	422.0	0.5	200.4
UAV (µmol/mmol creatinine)	<0.3	0.1	31.9	0.2	35.0

During the last six years, attacks have become infrequent (one to two times per year). She has been treated with on-demand non-steroidal anti-inflammatory drugs (NSAIDs) and short courses of prednisolone 1 mg/kg/day, with adequate control of the flares' symptoms and normal inflammatory markers during asymptomatic intervals. SAA regular screenings have been showing normal values. Serial repeated Autoinflammatory Diseases Activity Index (AIDAI) score results have been <9.

Case two

Case one's nine-year-old brother came to our attention after her referral. He had been diagnosed with recurrent viral respiratory infections and had normal growth and neurodevelopment. Since the first year of life, he presented recurrent fevers (lasting four to six days), upper respiratory symptoms, and maculopapular rashes (up to seven to eight episodes per year). Over the years, additional symptoms emerged during attacks: painful cervical lymphadenopathies, abdominal pain, occasional diarrhea, and polyarthralgia. Two episodes of mild orchiepididymitis were also reported. The parents had not looked for further medical help, since they had learned to manage attacks by similarity to his sister. Over time febrile episodes had become less frequent, and he was asymptomatic between episodes.

Investigations between episodes revealed a high IgA associated with normal IgD and normal ESR, CRP, and SAA. UAV excretion was also normal. During attacks, he presented elevated ESR, CRP, SAA, serum IgA and IgD, and also high UAV (Table 1). A genetic study confirmed MKD diagnosis since he carried the same MKD genotype as his sister. 

Over the last four years, he has been experiencing about two to three flares/year, lasting up to three days, and symptom control has been achieved mostly through NSAIDs and occasional on-demand steroids (two to three days). SAA values have also been normal. Serial repeated AIDAI score results have been <9.

## Discussion

HRFs represent a difficult and challenging diagnostic effort. We reported two siblings in whom, despite both presenting early infancy onset of recurrent febrile illness and characteristic associated symptoms, final MKD diagnoses were only established at 12 and nine years old, respectively. The identification of MVK gene variants in the eldest favored the earlier recognition of MKD in the youngest.

Owing to its wide spectrum of manifestations, with many being nonspecific and/or shared with other more frequent entities, a significant proportion of MKD patients present a long delay until its final establishment. Similarly to that seen in our patients, Van der Hilst et al. reported that in patients without a known family history, the median delay between disease onset and diagnosis was 9.9 years [6]. This long delay persisted despite the increasing availability for genetic testing over the last decade [6]. In addition, approximately one-third were initially misdiagnosed [6].

MKD is characterized by recurrent febrile episodes that start in early childhood, particularly during patients' first year of life, associated with lymphadenopathy (mainly cervical but almost all may be affected), gastrointestinal symptoms (abdominal pain, diarrhea, and vomiting), splenomegaly, polyarthralgia/arthritis and/or mucocutaneous manifestations, including rash and aphthous ulcers [3,6,8]. Prodromal symptoms such as chills, nasal congestion, sore throat, headaches, and others may precede impending attacks since they may be triggered by viral infections. These flares typically last for three to seven days and are separated by irregular asymptomatic intervals, commonly between four to eight weeks, but significantly variable even in the same patient and over time [8]. Although a trigger is not always apparent, factors like vaccinations have been reported as provocative [3], as seen in our eldest patient.

Some young patients may be misdiagnosed with PFAPA syndrome - presenting recurrent fevers lasting three to five days at roughly monthly intervals, with aphthous ulcers and cervical lymphadenopathy, positive response to steroids, and otherwise well outside attacks. PFAPA syndrome is clinically recognized by sudden periodic febrile episodes persisting for three to six days, with a spontaneous resolution, followed by an asymptomatic regular interval of three to six weeks and a subsequent repeated new cycle [2,9]. Pharyngitis, cervical lymphadenopathy, and/or aphthous stomatitis are the typical associated manifestations [9]. No infectious etiology is found and it presents a dramatic response to oral corticosteroids [9]. Arthralgias, mild gastrointestinal symptoms, and rash have also been described but are rarer [9]. According to Eurofever/PRINTO's clinical classification criteria, the absence of diarrhea, chest pain, skin rash, and arthritis present as favorable criteria [2]. In our patients, the early onset, irregular recurrent fever pattern, associated diarrhea, rash, and association with triggers were red flags for considering an HRF differential diagnosis, namely MKD.

However, although both patients presented many MKD-typical features, they also presented some atypical or rarer ones, which may have contributed to the diagnosis delay. Oral and genital aphthous ulcers may present in MKD, but the latter is a rarer manifestation. The eldest patient was referred to our unit after presenting *de novo *genital ulcers, which led to an unconfirmed Behçet's syndrome suspicion. In a previous case series of 103 HIDS patients, approximately 3% were imprecisely diagnosed with this latter syndrome [6]. Two episodes of mild orchiepididymitis were also reported in the younger one. Although being a feature of other HRFs, such as familiar Mediterranean fever, and tumor necrosis receptor-associated periodic syndrome (TRAPS), to our knowledge, orchitis has only been previously reported in one MKD patient [7].

Different clinical diagnostic and classification criteria have been proposed to help HRFs differentiation [2]. They have been lacking accuracy, especially due to some overlapping presentations, namely in younger children [9]. They also do not consider genetic studies, which are now an essential tool for the accurate recognition and classification of HRFs, in the presence of suggestive clinical and laboratory features [2,3]. Genetic diagnosis allowed the confirmation of the diagnosis in the index case, appropriate family screening, and genetic counseling [3]. On an individual basis, it improves investigation, treatment decisions, and appropriate screening of complications, such as amyloidosis [3]. Most MKD patients are compound heterozygotes, presenting a combination of biallelic variants of the MVK gene [2]. The identification of biallelic MVK variants in the eldest sibling enabled the suspicion and directed diagnosis of the youngest.

Although there is a tendency for the attacks to become less frequent and severe over time [6,9], associated HRFs systemic inflammation may result in progressive tissue/organ damage, morbidity, and increased mortality [3]. Reported MKD complications include growth delay, amyloidosis, joint contractures, abdominal adhesions, and hyperinflammation leading to macrophage activation syndrome [3,6]. It also presents an adverse impact on patients' quality of life, interfering with educational and employment achievements [6], underlying the importance of its early recognition and appropriate management.

MKD's treatment strategy should follow a treat-to-target approach, integrating patient-centered monitoring of disease activity, in order to find an individual treatment plan and target [5]. Treatment goals are low disease activity, assessed by clinical symptoms control and normalization of serum systemic inflammation markers, prevention of damage, and improvement of the patient's prognosis and quality of life [3,5]. The current MKD standard of care is subcutaneous IL-1 targeted biologic therapy [3,5]. Previous individual cases reported a response with tumor necrosis factor-alpha (TNF-α) or IL-6 inhibitors [5]. In milder disease phenotypes and/or cases of occasional attacks separated by asymptomatic and normal inflammatory marker intervals, patients can be managed with on-demand supportive treatment, namely NSAIDs and short-course glucocorticoids [3,9]. In our patients, NSAIDs and glucocorticoids have been effective. Their repeated AIDAI scores have been <9, and they have been presenting normal inflammatory markers, reflecting an inactive disease.

## Conclusions

Our reported cases underline how better HRF awareness is required for its early clinical recognition. Suggestive clinical features should trigger a pediatric rheumatology referral, which allows for an accurate diagnosis with an appropriate genetic study. This is paramount for its follow-up and therapeutic implications, improving patients' and families' quality of life, monitoring for complications, and also to detect affected family members and appropriate genetic counseling.
